# Web Ontologies to Categorialy Structure Reality: Representations of Human Emotional, Cognitive, and Motivational Processes

**DOI:** 10.3389/fpsyg.2016.00551

**Published:** 2016-04-25

**Authors:** Juan-Miguel López-Gil, Rosa Gil, Roberto García

**Affiliations:** ^1^Department of Computer Languages and Systems, University of the Basque CountryVitoria-Gasteiz, Spain; ^2^Department of Computer Science and Engineering, Universitat de LleidaLleida, Spain

**Keywords:** ontology, upper ontologies, emotion, cognition, motivation, Massive Open Online Courses

## Abstract

This work presents a Web ontology for modeling and representation of the emotional, cognitive and motivational state of online learners, interacting with university systems for distance or blended education. The ontology is understood as a way to provide the required mechanisms to model reality and associate it to emotional responses, but without committing to a particular way of organizing these emotional responses. Knowledge representation for the contributed ontology is performed by using Web Ontology Language (OWL), a semantic web language designed to represent rich and complex knowledge about things, groups of things, and relations between things. OWL is a computational logic-based language such that computer programs can exploit knowledge expressed in OWL and also facilitates sharing and reusing knowledge using the global infrastructure of the Web. The proposed ontology has been tested in the field of Massive Open Online Courses (MOOCs) to check if it is capable of representing emotions and motivation of the students in this context of use.

## Introduction

Ontology has been a field of philosophy since Aristotle and from its beginnings it has been characterized as a study of existence, a compendium of all there is in the world. Traditionally listed as a part of the major branch of philosophy known as metaphysics, ontology is the “branch of metaphysics that concerns itself with what exists” (Blackburn, 1996). Ontology deals with questions concerning what entities exist or may be said to exist, and how such entities may be grouped, related within a hierarchy, and subdivided according to similarities and differences.

Although, ontology as a philosophical enterprise is highly theoretical, the use of ontologies has expanded considerably in recent years in order to reflect different real-world concepts. One practical application is ontology engineering in information science and information technology, where an ontology is a formal naming and definition of the types, organized taxonomically, plus their properties and interrelationships that exist for a particular domain of discourse. In fact, an often-cited definition of ontologies in computer and information sciences is that “an ontology refers to an engineering artifact, constituted by a specific vocabulary used to describe a certain reality, plus a set of explicit assumptions regarding the intended meaning of the vocabulary words” (Guarino, 1998).

One of the most recent and potentially disrupting contributions of computer science to ontology has been moving them to the Web. In a Web Ontology, all defined concepts are identified using Web Uniform Resource Identifiers (URI), like:

http://sw.opencyc.org/concept/Mx4rvZLWaJwpEbGdrcN5Y29ycA

This URI corresponds to the concept for the field of study “Psychology.” This simplifies reusing this concept in other Web ontologies to just pointing to this URI from the ontology reusing the concept. For instance, if we want to define in our ontology the concept “Educational psychology,” we do not need to fully define it. We can define a subconcept that points using the *subClassOf* relation to the nearest one in OpenCyc ontology using its URI and just define the particularities of the new one. For instance:

http://mypsy.org/concept/EducationalPsychology

         ***subConceptOf***

http://sw.opencyc.org/concept/Mx4rvZLWaJwpEbGdrcN5Y29ycA

In order to make Web Ontologies really interoperable, basic relations like *subClassOf* have to be normalized so their meaning can be understood across different Web Ontologies. To this end, the World Wide Web Consortium (W3C) has standardized the Web Ontology Language (OWL) (Hitzler et al., 2009) for defining ontologies in web environments. OWL ontologies have formally defined meaning and provide classes, properties, individuals, and data values and are stored as shareable documents in the Internet. Web ontologies are formalized vocabularies of terms, often covering a specific domain and shared by a community of users. They specify the definitions of terms by describing their relationships with other terms in the ontology. Besides, they are especially useful when dealing with complex conceptual frameworks, as they provide an unambiguous representation of a conceptual framework and are expressive enough to make it possible to automate to a great extent sophisticated information processing services. Furthermore, ontology integration involves the creation of bridging modules between ontologies that accurately reflect the shared understanding of the semantic relationships between the different entities in the different ontologies (Hastings et al., 2014). The standard defines a set of primitives that constitute the building blocks to represent ontologies in the Web, like the *owl:subClassOf* relationship or the *owl:Class* concept. OWL also defines how these building blocks should be interpreted, their semantics, so all tools processing OWLs interpret them in a coherent way and automated reasoning is possible.

The hypothesis explored in this work is that it is possible to develop a functional Web ontology capable of linking categorical structures representing reality and the emotional, cognitive and motivational states people associates to these representations. The emotional state of people is important as it modifies their perception of the world, so it is important not only to adequately describe categorically structured ways of understanding the world around us, but also to describe the emotional, cognitive and motivational processes of people to understand how they perceive and interpret the world around them. Besides, both descriptions of reality and emotion, cognition and motivation can also be modeled by means of Web ontologies and all the knowledge shared in a common framework. This article describes the current state of development of an ontology that meets the previous goals, in which significant improvements have been introduced regarding the cognitive model in order to represent mechanisms that have proven to be relevant when it comes to recognize and generate emotions. Motivation has been introduced as a key element in generating emotional responses (Sartre, 1939; Lazarus, 1991).

In addition to explaining performed improvements, and in order to validate the proposal, it is also shown how the proposed ontology has been used in the field of Massive Open Online Courses (MOOC). Adequate representation of emotions and motivation is especially important in MOOCs in order to ensure their success among people who use them. A virtual agent was developed in order to gather information about how users interact with the system and assess how they felt and perceived everything surrounding the MOOC. Presented ontology was designed to link gathered user interaction data with the description of the MOOC environment, the concepts deployed and people interacting with the platform.

The rest of the paper is structured as follows. Next section describes materials and methods, i.e., the ontologies in the field of information sciences, the notion of upper ontologies and OWL, together with the ontology engineering methodologies applied to develop the contributed ontology. Section Results: the Emotions & Cognition Ontology presents resulting ontology for linking reality with its perception by human beings using emotion, cognitive and motivational processes, including information about existing models on how emotion, cognitive and motivational processes affect the perception of the surrounding world by individuals. Section Evaluation: Massive Open Online Courses presents the evaluation of this ontology in the context of MOOCs, where it allows determining what users perceived and felt while interacting with a MOOC. Finally, Section Conclusions outlines the conclusions.

## Materials and methods

Philosophers classify ontologies in various ways using criteria such as the degree of abstraction and field of application:

- **Upper ontology**: concepts supporting development of an ontology, meta-ontology. Also known as a top-level ontology or foundation ontology. It describes very general concepts that are the same across all knowledge domains.
- **Domain ontology**: concepts relevant to a particular topic or area of interest, for example, information technology or computer languages, or particular branches of science.
- **Interface ontology**: concepts relevant to the juncture of two disciplines.
- **Process ontology**: inputs, outputs, constraints, sequencing information, involved in business or engineering processes.

Recently, ontology has evolved a lot in the computer science and artificial intelligence fields. In these fields, an ontology is viewed as a formal, explicit specification of a shared conceptualization (Studer et al., 1998). “Formal” in the sense that it is an abstract model of a portion of the world and “explicit specification” because it is machine-readable and understandable. “Shared” implies that ontologies are based on a consensus and “conceptualization” that they are expressed in terms of concepts, properties, etc.

Ontologies were first used in Artificial Intelligence to facilitate knowledge sharing and reuse. Currently, their use is expanding to other disciplines related to information technologies and are starting to play an important role in supporting the information exchange processes, as they provide a shared and common understanding of a domain.

In computer science, ontologies are constructed using knowledge representation languages and logics. This allows automatic reasoning using the knowledge captured by ontologies. A great part of the meaning of expressions can be captured combining simpler concepts and conceptual relations. At the end, some preliminary set of fundamental concepts and relations is found. This set must have a rich semantic grounding in order to make powerful and valid automatic reasoning. Moreover, if it is shared among a great community, it may permit a great level of understanding.

As previously introduced, and also in the computer science field, the kind of ontologies providing fundamental concepts and relations are called upper ontologies. Upper ontologies, also known as foundational or top-level ontologies, try to formalize the more general concepts in our conception of the world and reality. These ontologies are fundamental to facilitate information and knowledge integration by automatic means. Thus, there have been many attempts to produce upper ontologies as detailed in Table 1.

**Table 1:** **Summary of the analyzed Upper Ontologies**.

**Ontology Name**	**Description**
Cyc	One of the biggest foundational ontologies is Cyc (Lenat, 1995), a project started in 1984 and that currently defines more than 239,000 concepts. A subset of that ontology has been released as an open ontology under the name OpenCyc and a more complete one for research purposes called ResearchCyc. The main value of this ontology is the enormous coverage it has gained over the years.
BORO Business Objects Reference Ontology	BORO is a reference ontology, designed for developing ontological or semantic models for large complex operational applications. It is based on a 4 Dimensional approach, where time is treated as another dimension, making it easier to capture change patterns. BORO also facilitates reuse because it is conceived as a framework to develop other ontologies under the same foundations.
UMBEL Upper Mapping and Binding Exchange Layer	It is an ontology of 28,000 reference concepts that maps to a simplified subset of the OpenCyc ontology. It provides near wide coverage of OpenCyc without the complexity of the knowledge representation languages used to define Cyc.
BFO Basic Formal Ontology	This ontology is specially conceived for the sciences, though it is kept really small because it does not enter into the particularities of any scientific domain. On the other hand, it is very generic because it incorporates both three-dimensionalist and four-dimensionalist perspectives on reality within a single framework.
DOLCE Descriptive Ontology for Linguistic and Cognitive Engineering	DOLCE (Gangemi et al., 2002) is an upper ontology with a clear cognitive bias, in the sense that it aims at capturing the ontological categories underlying natural language and human commonsense. Consequently, it is in many cases easier for non-ontology experts. For instance, the fundamental distinction between enduring and perduring entities, i.e. continuants and occurrents, is motivated by our cognitive bias.
SUMO Suggested Upper Merged Ontology	Many upper ontology initiatives were merged in the IEEE SUO effort (Standard Upper Ontology). The ontologies resulting from this effort, and specially the SUMO ontology (Suggested Upper Merged Ontology; Niles and Pease, 2001), are characterized by strong logical foundations that facilitate the implementation of sophisticated reasoning mechanisms on top of them. On the other hand, however, logical subtleties might make modeling more complex and time consuming.
UFO	UFO is a reference ontology of endurants, which is based on a number of different theories such as philosophy of language, formal ontology, linguistics, cognitive psychology and philosophical logics. Since UFO is a 3D ontology, it focuses less on processes and events. In order to deal with time and changes, additions to UFO have been made. It is called UFO-B, an ontology of perdurants.

Focusing on how upper ontologies can be used as Web ontologies, the previous example defining our custom concept representing “Educational Psychology” in terms of “Psychology” in OpenCyc can be represented using OWL as shown in Table 2. The first block, from lines 1 to 7, defines the other ontologies and parts of the OWL standard that are going to be reused. Then, lines 9–11 define the new ontology, including the URI that will be its global identifier (http://mypsy.org/concept/) and a human-friendlier label, “My Psychology Terms”. Finally, from line 13 to 16, the class for “Educational Psychology” is defined as a subclass of the “Psychology” class in OpenCyc. This example finishes with the closing mark in line 18, though a real ontology would include many more class definitions together with properties representing relationships among them, like the property “isTopicFor” relating concepts like the previous one and the class “Course.”

**Table 2:** **Example of Web Ontology using the Web Ontology Language (OWL) standard**.

1	<!DOCTYPE Ontology [< !ENTITY cyc “http://sw.opencyc.org/concept/">]>
2	<rdf:RDF
3	xmlns = "http://mypsy.org/concept/"
4	xmlns:cyc = "http://sw.opencyc.org/concept/"
5	xmlns:owl = "http://www.w3.org/2002/07/owl#"
6	xmlns:rdf = "http://www.w3.org/1999/02/22-rdf-syntax-ns#"
7	xmlns:rdfs = "http://www.w3.org/2000/01/rdf-schema#">
8	
9	<owl:Ontology rdf:about = "**http://mypsy.org/concept/**">
10	<rdfs:label>My Psychology Terms </rdfs:label>
11	</owl:Ontology>
12	
13	<owl:Class rdf:ID = "**EducationalPsychology**">
14	<rdfs:label>Educational Psycology </rdfs:label>
15	<rdfs:subClassOf rdf:resource = "&cyc;Mx4rvZLWaJwpEbGdrcN5Y29ycA"/>
16	</owl:Class>
17	
18	</rdf:RDF>

From this Web Ontology, an automated reasoner processing it using OWL semantics can infer, without any additional knowledge, that it makes sense to recommend courses about “Educational Psychology” when someone is looking for courses about “Psychology,” because the latter includes all instances of the former from a logic standpoint. Moreover, the reasoner can follow the URI to OpenCyc to retrieve additional information about this class, for instance labels in different languages or how it is related to other concepts.

### Modeling cognition, motivation, and emotion using web ontologies

One of the great challenges of artificial intelligence has been to conceptualize a model of human behavior using technological agents. There are different theories combining emotion and cognitive concepts. Scherer et al. (2010) classify them as discrete, dimensional and appraisal theories of emotion.

Focusing on the models of emotions used in this work, appraisal theories can be translated as evaluation or estimate. They are seeking to detail the underlying mental processes related to elicitation of emotions. That is to say, reflects the person-environment relationship, do not take into account only one aspect. This relationship is characterized by size (appraisal variables). A possible example would be: Is this a desirable event or a desired objective? Who caused it? Or do you expect? The results are mapped on emotions. Some derived models describe in detail how the resulting emotions influence individual cognitive and behavioral responses. Concepts from traditional artificial intelligence BDI models (beliefs, desires and intentions) are mapped to the dimensions from the appraisal theories. The computer models used are the Emotion and Adaptation models.

**Figure 1:**
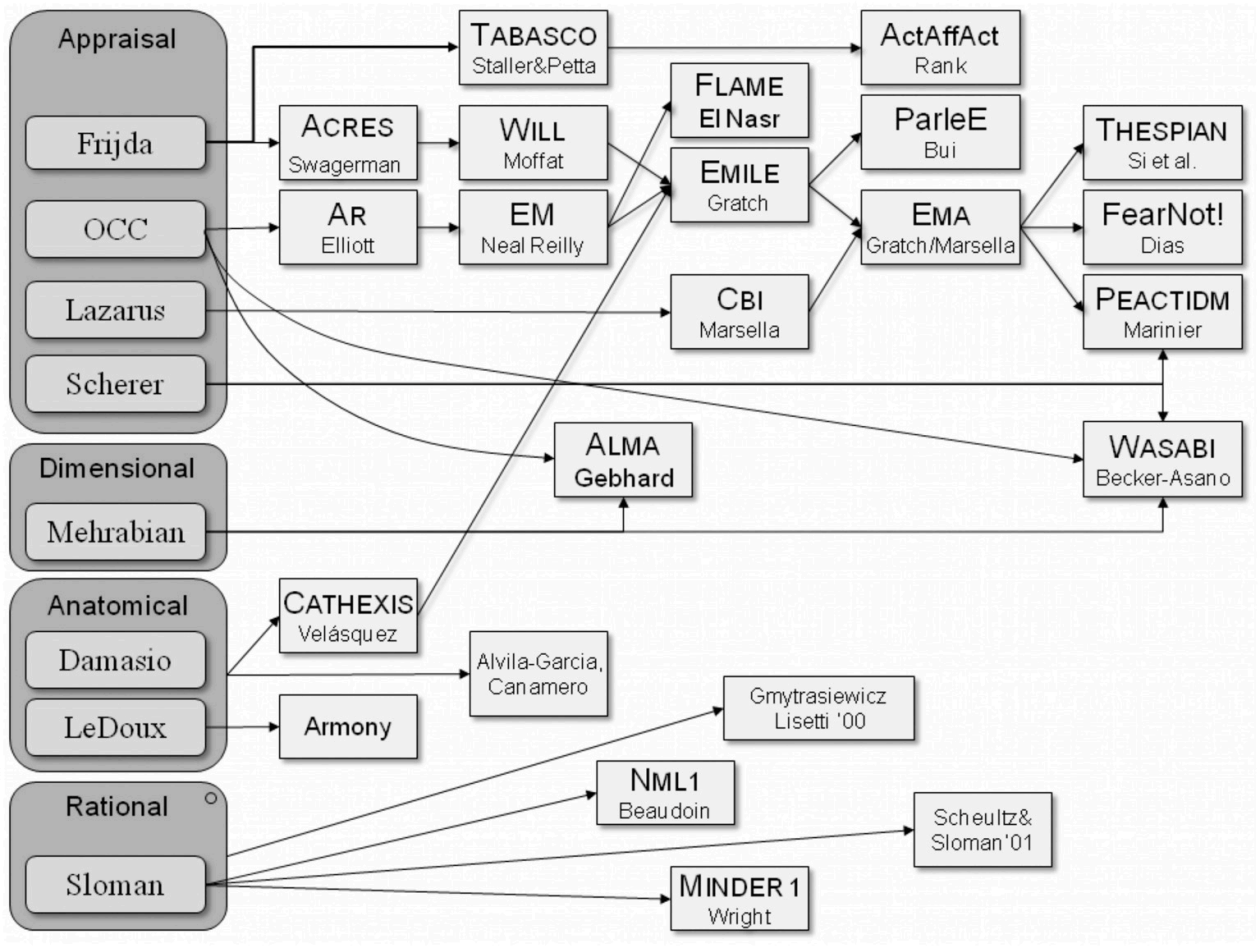
**Emotional models in artificial intelligence (Scherer et al., 2010)**.

Each of them has resulted in more refined theories on emotional computer models, as shown in Figure 1. The following issues are aspects of these models, we see that sometimes are hybrids as they include aspects of two theories in order to show that all are included in the ontology proposal (see next section). One of the referenced models, ALMA (A Layered Model of Affect), is referred because it is a hybrid model (Gebhard, 2005) as also shown in Figure 1.

Anatomical models emphasize the neuronal basis, so that we can talk about different ways (low-road, high-road) for the elicitation of an emotion, so that these models focus on low level of perceptual-motor tasks encoding a dual process as seen emotion (perception ontology). In the first case, there is a rapid and automatic response while, secondly, a slower response comes from reasoning processes (cognitive processes in the ontology). Consequently, affect models allow modeling cognitive or behavioral consequences, while (Chiew and Braver, 2011) also explores the motivational part related to cognitive and emotional processes.

Cognitive neuroscience aims at mapping mental processes onto brain function, which begs the question of what “mental processes” exist and how they relate to the tasks that are used to manipulate and measure them. Poldrack et al. (2011) proposed that cumulative progress in cognitive neuroscience required a more systematic approach to representing the mental entities that are being mapped to brain function and the tasks used to manipulate and measure mental processes. As a result, Cognitive Atlas^1^, an ontology that characterizes the state of current thought in cognitive science was developed.

The Emotions Ontology (Hastings et al., 2014) is an ontology of emotion based on the BFO (Basic Formal Ontology) presented in Section Materials and Methods. Like BFO, this ontology is specially intended for the scientific domain and particularly to the biological sciences and human health. For instance, in combination with biology ontologies also based on BFO, it is capable of representing neurotransmitters and their influence in emotional processes and responses. Moreover, due to being based on BFO, it is a sophisticated ontology with strong logical foundations capable of modeling complex logical expressions. However, from our perspective, this makes it more difficult for people without an ontology modeling background.

### Web ontology engineering

Starting from the previous building blocks (ontologies, technologies, theories, etc.), and in order to generate a consistent ontology that satisfies the requirement, an ontology engineering methodology has been applied. The Methontology methodology (Fernández-López et al., 1997) has been chosen because it provides guidance for ontology development process but also for other support and management activities. Moreover, it is extensively based on “classical” software engineering methodologies and this fact makes it easier to learn and apply for people with some software engineering experience.

Methontology proposes some ontology management activities, which include scheduling, control and quality assurance. There are also ontology support activities, which are performed at the same time as the development-oriented activities, namely: knowledge acquisition, evaluation, reuse (merging or aligning other ontologies), documentation and configuration management. These are all support activities while the main ontology creation work is performed in development process.

The development process is composed by the following phases: specification, conceptualization, formalization, implementation and maintenance. The specification phase corresponds to the pre-development aspects, where the development requirements are identified. The maintenance phase is a post-development activity, which is performed once the ontology is developed. During the conceptualization activity, the domain knowledge is structured as meaningful models. Moreover, if a formal language is used to build the model, it is possible to automate the formalization and implementation activities. In our case, as OWL is a formal language, we can benefit from this feature and existing ontology development environments implementing it, like Protégé (Musen, 2015). Moreover, as it is detailed in Section Evaluation: Massive Open Online Courses, it is also possible to use automated logical reasoners to check the consistency of the resulting ontology.

## Results: the emotions & cognition ontology

First of all, for the development of our ontology Emotions & Cognition Ontology, the chosen knowledge representation for the contributed ontology is the Web Ontology Language (OWL), which also facilitates sharing and reusing knowledge using the already global infrastructure of the Web. This, compared to existing pre-Web ontologies, facilitates sharing and also reusing existing ontological frameworks as it is detailed next.

Our approach has been to reuse as much as possible existing ontologies, especially upper ontologies and other more specific ones related to cognition and emotion. This approach reduces the cost of developing an ontology but also strengthen it because it is based on more solid foundations, provided by already proven and widely used ontologies.

The first choice has been to reuse the upper ontology Cyc. The main motivation has been to benefit from its wide coverage. This way, it is usually possible to find amongst the 239,000 concepts it provides the required ones to model the real situations for which we want to capture their perception taking into account cognitive, motivational and emotional factors.

Basically, whenever a particular term is needed, we can search Cyc, locate the relevant concept based on the provided descriptions and relations to other concepts and, finally, refer to that concept using its reference. This is facilitated by the fact that we are using a Web ontology and that OpenCyc is also available as in that form.

However, our experience showed that beyond providing a lot of base concepts where we can root the ones introduced by our ontology, OpenCyc was too normative and rigid when trying to model the glue among these concepts that capture the parts of reality we are interested in modeling. OpenCyc is based on a strong use of logic geared toward automated reasoning that requires a profound knowledge and effort. We required a modeling approach less abstract and more similar to what we were trying to capture, human cognition.

Based on these requirements, the clear choice was DOLCE, whose aim is precisely to capture the ontological categories underlying natural language and human common sense. DOLCE does not commit to strictly referentialist metaphysics related to the intrinsic nature of the world like 4D ontologies do. Rather, the categories it introduces are thought of as cognitive artifacts, ultimately depending on human perception, cultural imprints and social conventions. In this sense, they intend to be just descriptive notions that assist in making already formed conceptualizations explicit.

For instance, the distinction between enduring and perduring entities is simplified in DOLCE to the relation of participation: an endurant “lives” in time by participating in some perdurants. For example, a person, which is an endurant, may participate in a discussion, which is a perdurant. A person's life is also a perdurant, in which a person participates throughout its all duration. Using this approach, we have rooted the contributed ontology on the fundamental terms provided by DOLCE. This facilitates the process of modeling real world situations and their perception. On the other hand, given the limited scope of DOLCE, when specific terms for concepts like “Psychology” are needed to build a representation, then we look into OpenCyc and refer to them using their URIs.

DOLCE is also a Web ontology. Consequently, this approach makes it easy for agents to process the new ontology, as they just need to follow its links in order to retrieve additional facts about the reused concepts. The vision of this approach is that, this way, ontologies can grow and evolve organically through the web in an unrestricted and self-organized way, like the Web did with great success. Another example of this vision is the rhizome metaphor proposed by Deleuze and Guattari (1987).

In addition to these advantages, other key features of Emotions & Cognition Ontology are:

1. The underlying conceptual model, implemented by the ontology, is independent from any specific emotion theory. It provides a set of building blocks (concepts) that are selected and combined as required in order to capture the subtleties of a particular model of cognition or emotion.
2. The ontology is capable of dealing with different emotion communication modalities. The model includes generic concepts like *Sensor* or *Emotion Expression System*, which are then refined to specific kinds, like biological (eye, taste…) or artificial sensors (camera, microphone…).
3. Reality is represented by means of different ontologies, which are used combined with proposed ontology to represent the world around us.
4. Reality models are based on the notion of context, which provides flexible and accurate ways of modeling situations, as detailed next.

### Fundamental building blocks

The previous features are based on the use of the DOLCE upper ontology, which provides the fundamental building blocks like the *Context* or *Sensor* concepts. Consequently, our proposed ontology extends the DOLCE upper ontology and particularly the *Description* and *Situation* concepts. *Perception* generates *Descriptions* that represent *Situations*, configurations of the real world. These *Descriptions* may trigger and be associated with emotions.

Another fundamental feature of the proposed ontology is that it does not commit to a particular emotions theory. For instance, an example of emotions ontological modeling might be just to represent using an ontology and as a taxonomy the categorical theory of emotions by Ekman (1984). However, this limits the proposed ontology just to the application of this particular emotions model.

Consequently, even from the initial steps of defining an ontology for describing just emergent emotions (López et al., 2008), Emotions & Cognition Ontology has been planned as emotion theory agnostic model. Thus, it is capable of providing the required mechanisms to model reality and associate it to emotional responses, but without committing to a particular way of organizing these emotional responses. This approach is improved in this new proposal as we have now consolidated the ontological foundations provided by DOLCE and Cyc, and other resources reused to facilitate *Descriptions* modeling like FrameNet. Moreover, the ontology includes now the appraisal aspects described in Section Results: the Emotions & Cognition Ontology.

The flexibility of Emotions & Cognition Ontology is due in great measure to the inclusion of the generic concepts reused from OpenCyc and specially DOLCE, combined with the mechanisms that the ontology provides to model the interactions between an agent and its environment, something that is fundamental in emotion theories based on appraisal.

**Figure 2:**
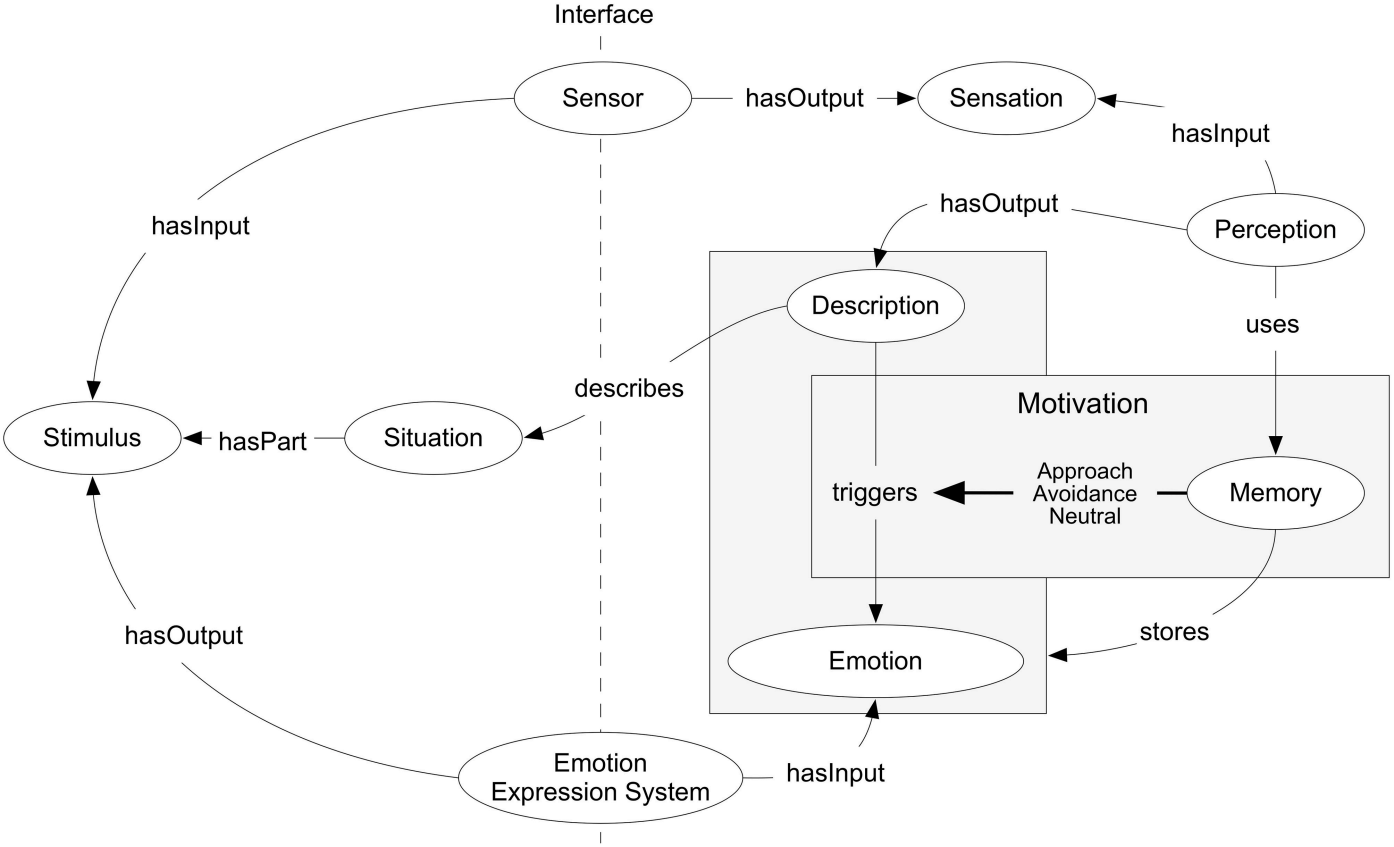
**Emotions & Cognition Ontology overview**.

In this regard, from DOLCE we reuse the concepts of *Description* and *Situation*, which constitute the basic building blocks to model the relationship among agents and their environment, cognitive processes and motivation. The cognitive process of *Perception*, as shown in Figure 2, generates *Descriptions*, which are representations made by the agent holding that cognitive process of the perceived *Situations*, configurations of the real world identified by the agent. These representations, the *Descriptions*, are what the agent associates to emotions as a result of its cognitive and motivational processes.

The fundamental building blocks also include other cognitive process and related aspects, which can be used and combined depending on the particular emotion and cognition theories to work with, and the required detail level. As it is shown in Figure 2, the ontology also includes a generic concept *Emotion* that can be directly extended and refined. This is usually enough when working with dimensional theories of emotion. Additionally, if theories based on appraisal are considered, the ontology also provides mechanism to model context using concepts like *Perception* or *Memory*, combined with the separation between *Environment* and *Agent*.

The interface between the *Environment* and the *Agent*, from an emotions point of view, is defined by *Sensors* and the *Emotion Expression System*. As the ontology defines *Agent* as a generic concept, which includes both human agents and artificial ones, the sensors include human senses but also artificial sensors. The same applies to the emotion expression systems under consideration.

On the other hand, the Environment corresponds to the agent “reality,” a real world or a virtual one in which virtual agents interact. The latter might include the Internet or a particular Web application like a specific social network. In this case, the Web application is what determines the available sensors and emotions expression systems, for instance emoticons.

Finally, in the *Agent* side, in addition to *Perception*, the ontology defines other cognitive processes like *Memory* and *Motivation*. These additional cognitive processes allow modeling the key aspects of the appraisal factors, from previous agent experiences that define its beliefs to the desires and intentions that configure its motivations.

All the cognitive processes have been linked to the main cognitive science ontology identified in Section Results: the Emotions & Cognition Ontology, Cognitive Atlas. For instance, *Memory* has been linked to the corresponding concept in Cognitive Atlas *memory*^2^, which also provides access to specific kinds of memory if such level of detail is required, like *context memory* or *emotional memory*. Other relevant concepts from Cognitive Atlas the ontology is linked to are: *perception*^3^ or those related to *Motivation*^4^.

*Memory* stores past associations of *Descriptions* and the *Emotions* triggered by the corresponding *Situations*. These memories are fed into *Motivation*, which matches the current *Description* to past memories. If the match strength is low, because the corresponding situations have little in common and consequently the associated *Descriptions* too, the motivation is *Neutral Behavior* and the effect of motivation on the triggered *Emotion* is low or inexistent.

On the contrary, if the match is high, because the *Descriptions* of the current situation and the past one are similar, then the motivation is non-neutral. In this case the effect on emotion triggering might be positive or negative, positive if the *Memory* associated the matched *Description* to a positive *Emotion*, or negative if it was a negative one. The former corresponds to an *Approach* behavior and the latter to an *Avoidance* one, from a motivational point of view.

What constitutes a positive or negative *Emotion*, and its effect on *Motivation*, will depend on the particular emotion theory to be applied and on the available mechanisms to characterize emotions, as it is detailed in the practical scenario described in Section Conclusions.

Once the previously introduced ontological building blocks have been described, the next subsection details how the main building blocks to model reality, i.e., *Descriptions*, are defined. These descriptions capture the aspects of reality considered relevant by the agent and link them to emotional responses.

### Modeling descriptions of reality

In order to cope with the enormous range of different situations that might need to be associated with emotions, their descriptions are modeled using concepts from OpenCyc, as previously introduced. In addition to OpenCyc concepts, we have also included terms from FrameNet (Scheffczyk et al., 2008). This is not an upper ontology but a big lexical database, with more than 10,000 word senses, structured following Frame Semantics (Fillmore, 2006).

Frames fit really well with situations modeling as they try to explain words meaning by building a description of a type of event, relation, or entity and the participants in it. This way, DOLCE provides the ontological foundations, FrameNet the glue to structure situations modeling and OpenCyc the anchors to the specific concepts involved in situations and descriptions modeling. Section Conclusions provides examples of how these ontologies are reused to this end. These examples will use the STUDYING frame, which is presented in Table 3.

**Table 3:** **FrameNet description of the frame STUDYING**.

**STUDYING**
**Definition**: A Student enrolls and then remains at an Institution for the purposes of education within a Subject. They may receive instruction from a particular Teacher at the Institution.
**Frame Elements**
**Core:**
**• INSTITUTION**: an educational establishment, such as a school or college.
**• STUDENT**: one who receives instruction from a Teacher or Institution.
**• SUBJECT**: the area of knowledge or skill which is taught to a Student.
**• TEACHER**: one who instructs a Student in some area of knowledge or skill.
**Non-Core:**
**• CO-PARTICIPANT**: an entity that participates in a coordinated way in the event.
**• DEPICTIVE**: a state which describes the Student during the process of study.
**• DURATION**: the amount of time for which the process of study is ongoing.
**• EXPLANATION**: the reason for which the Student studies the Subject.
**• INTENDED ACADEMIC CREDIT**: the Student's motivation for studying.
**• LEVEL**: identifies the Level of a student in his/her education.
**• MANNER**: the manner of studying that the Student has.
**• PLACE**: the Place is the location within which the studying takes place.
**• TIME**: the time when the student is studying.

For a complete list of all the concepts defined in Emotions & Cognition Ontology, it is available online as a Web Ontology^5^. In the next section, this ontology is put into practice in a particular scenario, online education.

## Evaluation: massive open online courses

The aim of this section is to evaluate Emotions & Cognition Ontology in the context of a real use case. Web ontologies can be evaluated from a purely logical standpoint using a reasoner capable of processing OWL. There are many OWL reasoners available and we have used one of them, Pellet (Sirin et al., 2007) to validate the consistency of the ontology. However, the consistency of an ontology is just a lightweight evaluation. We have the guarantee that it is not going to generate contradictory conclusions but we don't know if it is going to be capable of modeling the required knowledge and producing the expected conclusions.

For this kind of evaluation, it is necessary to put the ontology in use in a real or simulated scenario. We have applied previous versions of this ontology to gather emotional common sense (Gil et al., 2015) and in the context of tangible user interfaces (López-Gil et al., 2014). More recently and as detailed next, we have also started to apply the enhanced version including cognition in the context of online education and Massive Open Online Courses (MOOCs).

MOOCs are a recent development in distance education that allow the participation of a big amount of users and that can be accessed using the Internet. They have become popular since 2012, when some courses vendor platforms such as Coursera, in which prestigious universities participated, emerged. In addition to course materials, such as videos with lectures, readings or sets of problems, MOOCs also provide interactive forums and online communication tools to promote interaction between students and teachers.

Despite their popularity, the MOOCs also have disadvantages and associated challenges, including that relying on user-generated content can generate a chaotic learning environment, necessary knowledge of the online platform to make appropriate use of it, the time and effort required by the participants, the difficulty of controlling the course trajectory once it has been released due to the amount of different students and self-regulation of users to obtain the expected educational benefit. All these aspects are strongly influenced by the characteristics of the users and their expectations, which may result in different emotional and motivational states depending on how the course is elapsing.

In this type of systems motivation is especially important. It is an important factor to improve the performance of students and to improve the ratio of pupils that successfully complete the courses that are enrolled in. In addition, modeling the emerging emotions that a person expresses is also important in such environments in order to learn how they are feeling.

Emotions & Cognition Ontology can represent different emotions expression systems that can be used in these environments as a basis for recognizing the emotions of users and also to analyze their motivation. In the case of MOOCs, the agent expressing emotions is the human being, so different emotions expression systems are considered, including natural language, speech, facial expressions, and even galvanic skin response, brain activity, heart rate, or blood pressure.

However, before we can start representing emotional responses, we need to model the real world situations to which they are associated. As presented in Section Evaluation: Massive Open Online Courses, we will use DOLCE *Descriptions* as the representations of the real world *Situations*. Moreover, we are going to use FrameNet frames and OpenCyc concepts to provide the required level of detail to these *Descriptions*.

In our scenario, one frame that is particularly relevant is the one shown in Table 3 in the previous section, the frame STUDYING. We will use this frame to illustrate how the ontology can be used to model a *Description*. For instance, a situation in this scenario might be “*The second grade student Stuart Adams has been studying educational psychology online course for 2 h today.*” The *Description* for this situation is based on the STUDYING frame, where the frame elements are filled with different parts of the situation as follows:

**[STUDYING]**→**[STUDENT: Stuart Adams]**

                    →**[LEVEL: second grade]**

                    →**[SUBJECT: educational psychology]**

                    →**[MANNER: online course]**

                    →**[DURATION: 2 hours]**

                    →**[TIME: today]**

The idea is that, given the previous *Situation*, an agent perceives it through its sensors, sense in the case of a human agent, and its cognitive processes generate the corresponding *Description*, the representation that the agent builds for its environment. With the ontology, and for the online education scenario, the objective is to try to mimic this behavior so we can first model the context of the student being monitored, and then associate an emotional response so we can improve the student experience.

However, before we connect the *Description* to emotions, we can detail it further, going beyond the use of FrameNet frames. The *Description* can be enriched with concepts from Web ontologies like OpenCyc, which help defining, for instance, what “educational psychology” refers to. It might be the case this particular concept is not present in OpenCyc, we can then define it as we did in the example in Section Evaluation: Massive Open Online Courses as a subconcept of “psychology,” which is defined in OpenCyc as shown in Table 4.

**Table 4:** **OpenCyc definition for the concept Psychology**.

OpenCyc Individual: **Psychology**
Unique ID: [ Mx4rvZLWaJwpEbGdrcN5Y29ycA ]
English ID: [ Psychology ]
The **FieldOfStudy** of psychology.
• Instance of: **FieldOfStudy**
• Wikipedia: http://en.wikipedia.org/wiki/Psychology
• Same as:
• http://umbel.org/umbel/sc/Psychology
•http://data.linkedmdb.org/resource/film_subject/369
•http://dbpedia.org/resource/Psychology

This way, the frame element for the previous description can be further detailed to:

→**[SUBJECT:** http://mypsy.org/concept/EducationalPsychology

    ***subConceptOf***

http://sw.opencyc.org/concept/Mx4rvZLWaJwpEbGdrcN5Y29ycA**]**

In this case, the refinement makes it less ambiguous what Stuart Adams is studying, specially from the point of view of an artificial agent who has to monitor and respond to his emotional responses, for instance to adjust the learning pace of this particular student. It might be the case that the agent does not have any particular knowledge about the concept EducationPsychology, but it can at least follow its definition as a subconcept of the OpenCyc concept for Psychology, and from there learn that it is a field of study also defined in other Web ontologies such as the DBPedia, which is the Web ontology version of Wikipedia.

This refinement allows the agent to recognize that the current subject is related to previous ones he has also studied, which appeared to be especially frustrating for the user given the records of existing descriptions and emotional responses. Consequently, it might be convenient to anticipate and adjust the pace to improve the learning experience in this case. This is supported by another cognitive process also modeled by the ontology, *Memory*. The MOOC agent can use these sensitive memories to represent past *Descriptions* and their associated *Emotions*.

This way, we can use *Memories* to also model *Motivation* and its influence on the emotional response to the current *Situation*. The proposed approach is based on matching the *Description* for the current situation with *Descriptions* associated to past *Memories*, which in the case of the MOOC agent will be stored by the agent to try to anticipate the motivations of the student.

As mentioned in the case of the Educational Psychology subject, the matching does not need to be direct. Though the current *Description* refers to the concept *EducationalPsychology* and there are no previous memories with descriptions referring to this same subject, it might be the case that there are past memories that are associated to subject related to OpenCyc concept *Psychology* like *EducationalPsychology*.

Therefore, we need a matching algorithm that takes into account, for instance, the amount of concepts that the compared descriptions share. However, this algorithm can be further sophisticated to take into account structure and semantics (Gallagher, 2006). In any case, what is needed is a mechanism to derive for each memory its behavior associated to *Motivation*.

First of all, if it is mainly a *Neutral Behavior*, for instance if the current description and the past one just share a small amount of concepts. If the behavior is not neutral, then two behaviors can be derived: *Approach* and *Avoidance*. The former, if the recorded emotional response for the memory had at least a positive valence, is even clearer if the arousal was also positive. On the other hand, the behavior anticipated by *Avoidance* would be clearer if the response had a negative valence.

Thus, the MOOC agent will use the combined set of behaviors for the non-neutral motivations to try to anticipate the motivation of the student for the current situation. For instance, if there is *Memory* for a subject is related to OpenCyc *Psychology* with an emotional response characterized by a positive valence and arousal, as registered by available sensors, the agent can infer a non-neutral motivation of *Approach*. This information can be then used to adjust the student pace.

**Figure 3:**
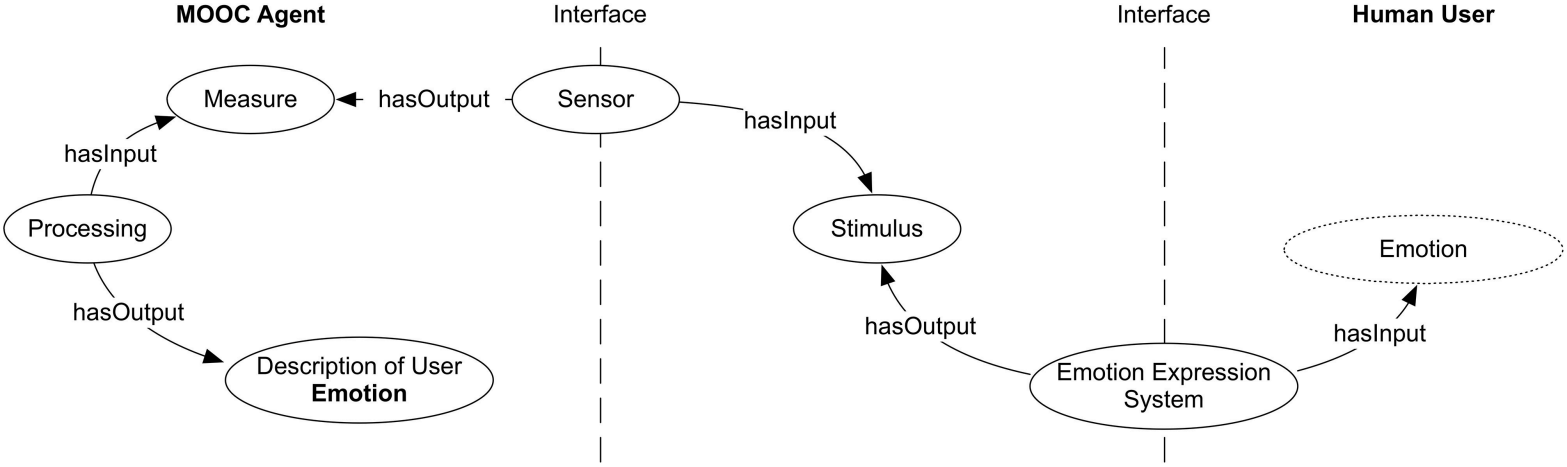
**Emotions & Cognition Ontology model of the interaction between the student (Human User) and the artificial agent (Intelligent Emotion-aware Agent)**.

The previously mentioned sensors, used by the agent, can be also modeled using Emotions & Cognition Ontology. The agent uses them to monitor emotions expression systems of students, as shown in Figure 3, where the agent is displayed in the left side and the student on the right. The environment in this case, represented in the center of the figure, consists of the MOOC system itself and the physical environment in which the student interaction is carried out.

The range of sensors available to the MOOC agent will depend on the devices available to each student. Our current experimentation setting includes the following devices:

- **Microphone:** Captures vocal parameters and natural language.
- **Keyboard:** Captures the natural language.
- **Webcam:** Captures facial expressions.
- **Wristband:** Wristband with sensors to capture the galvanic skin response, heart rate and blood pressure.
- **Neuroheadset:** Headset with sensors to capture brain activity by gathering data from EEG channels.
- **Eye Tracker:** Captures the focus of the user within the given user interface.

All the information provided by the sensors feeds the artificial agent. With the Emotions & Cognition Ontology, we are able to set what the agent needs at the conceptual level, which is the aim of this paper. From now on, we need to face what can be called the emotion semantic gap between the signals captured by the sensors the agent includes and the conceptual representation of the recognized emotion at the conceptual level, i.e., as an *Emotion*.

We have already tested the feasibility of this approach for some of the sensors in our experimentation setting. For instance, we have used a combination of techniques and applications to infer the emotional state of the student and render it using valence and arousal dimensions, following the PAD model mentioned in Section Results: the Emotions & Cognition Ontology. The electro-physiological experiments were carried out according to the principles of the declaration of Helsinki and approved by the ethics committee on clinical research of the Arnau de Vilanova University Hospital. With appropriate software for processing EEG and other electrophysiological data, such as EEGLab^6^, we process the neuroheadset signal and derive the arousal from the EEG signal, while the valence is derived from a wristband. These values are fed to the MOOC agent so it can associate them to the *Description* of the *Situation* the student is faced at that particular moment. A sample dataset based on the Emotions & Cognition Ontology for the MOOC scenario presented in this section is available online^7^.

## Conclusions

This article presents an ontology for linking reality with its perception by human beings. As the emotional state of people is important as it modifies their perception of the world, it is important not only to adequately describe categorically structured ways of understanding the world around us, but also to describe the emotional, cognitive and motivational processes of people to understand how they perceive and interpret the world around them. Besides, as both descriptions of reality and emotion, cognition and motivation are modeled by means of ontologies, all this knowledge is shared in a common framework.

It is also shown how proposed ontology has been used in the field of MOOCs, environments where adequate representation of emotions and motivation is especially important to ensure its success among people who use them. Testing the ontology in real scenarios has allowed the validation of one of the main aims of the ontology: it is relatively simple to apply even for non-experts in ontology modeling.

Additionally, the rest of the intended contributions detailed in Section Fundamental Building Blocks have been also addressed. First, as the scenarios are based on different theories of emotion, it has been possible to test that the Emotion & Cognition Ontology provides the required building blocks to accommodate these different views, from discrete or dimensional theories considered in the context of Tangible User Interfaces (TUI) (López-Gil et al., 2014) to those based on appraisal, as illustrated in this paper. The scenarios have also shown that the ontology is capable of dealing with different emotion communication modalities, from physical sensors and emotion expression systems, available in the case of TUIs, to virtual ones like in the case of MOOCs.

On the other hand, the approach based on Web Ontologies has facilitated reusing many different ontologies from upper ontologies like DOLCE or OpenCyc to the reuse of frames from FrameNet, which has considerably reduced the modeling effort. The latter has also highlighted the advantages of including the notion of context in the core of the ontology through DOLCE's Descriptions and Situations, which have been smoothly connected with the notion of frames to facilitate Descriptions modeling.

All these findings corroborate the contribution beyond existing emotions ontology, specially comparing to the Emotions Ontology (Hastings et al., 2014), which is the main ontology in this field and was introduced in Section Results: the Emotions & Cognition Ontology. Emotions Ontology is more sophisticated than the proposed one, as it is based on an upper ontology that makes use of logic formalisms to enable elaborate reasoning. However, this introduces too much unnecessary complexity when working with simpler emotion theories or scenarios where just a simple modeling of emergent emotions is required. Moreover, Emotions Ontology is quite tied to a vision of emotion based on the concept of appraisal.

As technology advances, different types of sensors are available to gather information about people's emotions, cognitive processes and emotions, such as EEG, heart rate, electrodermal activity, facial expressions or speech. Still, such information is not enough by itself to determine such complex aspects and must be considered in the frame of established models and theories. The ontology formalizes a common view about how theories and models are mapped, which are then used to facilitate data integration. If these mappings would not be provided by the ontology, the semantic gap would remain and interoperability among ontology components would be seriously compromised.

Neuroscience is advancing day by day in the knowledge of how humans manage emotions. There are many emotional computing models that relate the abstract concepts that included in the ontology. However, in the not too distant future, emotions will not be restricted to humans, as it seems that machines and virtual agents in general will also be able to recognize and synthesize emotions. Exposed line of work aims to set out a general framework for all kinds of emotional interactions, including the ones with such emotion-aware devices and virtual agents.

## Author contributions

All authors listed, have made substantial, direct and intellectual contribution to the work, and approved it for publication.

### Conflict of interest statement

The authors declare that the research was conducted in the absence of any commercial or financial relationships that could be construed as a potential conflict of interest.

## Footnotes

^1^http://www.cognitiveatlas.org/

^2^*Memory* in Cognitive Atlas, http://www.cognitiveatlas.org/concept/memory

^3^*Perception* in Cognitive Atlas, http://www.cognitiveatlas.org/concept/perception

^4^*Motivation* category in Cognitive Atlas, http://www.cognitiveatlas.org/concepts/category/Motivation

^5^http://rhizomik.net/ontologies/emotions&cognitionontology/

^6^http://sccn.ucsd.edu/eeglab/

^7^Sample dataset for the MOOC scenario: http://rhizomik.net/ontologies/2015/12/mooc-sample.ttl">
